# Cancer Glycolytic Dependence as a New Target of Olive Leaf Extract

**DOI:** 10.3390/cancers12020317

**Published:** 2020-01-29

**Authors:** Jessica Ruzzolini, Silvia Peppicelli, Francesca Bianchini, Elena Andreucci, Silvia Urciuoli, Annalisa Romani, Katia Tortora, Giovanna Caderni, Chiara Nediani, Lido Calorini

**Affiliations:** 1 Department of Experimental and Clinical Biomedical Sciences “Mario Serio”, University of Florence, 50134 Florence, Italy; jessica.ruzzolini@unifi.it (J.R.); silvia.peppicelli@unifi.it (S.P.); francesca.bianchini@unifi.it (F.B.); e.andreucci@unifi.it (E.A.); 2 PHYTOLAB (Pharmaceutical, Cosmetic, Food Supplement Technology and Analysis)-DiSIA, Scientific and Technological Pole, University of Florence, 50019 Sesto Fiorentino (Florence), Italy; silvia.urciuoli@gmail.com (S.U.); annalisa.romani@unifi.it (A.R.); 3 NEUROFARBA Department, Pharmacology and Toxicology Section, University of Florence, 50139 Florence, Italy; katia.tortora@unifi.it (K.T.); giovanna.caderni@unifi.it (G.C.); 4 Center of Excellence for Research, Transfer and High Education DenoTHE University of Florence, 50134 Florence, Italy

**Keywords:** olive leaf extract, oleuropein, Seahorse analysis, cancer metabolism, glycolytic markers

## Abstract

Oleuropein (Ole), the main bioactive phenolic component of *Olea europaea* L. has recently attracted the scientific attention for its several beneficial properties, including its anticancer effects. This study is intended to investigate whether an olive leaf extract enriched in Ole (OLEO) may counteract the aerobic glycolysis exploited by tumor cells. We found that OLEO decreased melanoma cell proliferation and motility. OLEO was also able to reduce the rate of glycolysis of human melanoma cells without affecting oxidative phosphorylation. This reduction was associated with a significant decrease of glucose transporter-1, protein kinase isoform M2 and monocarboxylate transporter-4 expression, possible drivers of such glycolysis inhibition. Extending the study to other tumor histotypes, we observed that the metabolic effects of OLEO are not confined to melanoma, but also confirmed in colon carcinoma, breast cancer and chronic myeloid leukemia. In conclusion, OLEO represents a natural product effective in reducing the glycolytic metabolism of different tumor types, revealing an extended metabolic inhibitory activity that may be well suited in a complementary anti-cancer therapy.

## 1. Introduction

The Pasteur effect describes the inhibition of glycolysis by oxygen, through the inhibition of phosphofructokinase-1, the most important controlling enzyme of glycolysis, by ATP and citrate [1]. Otto Warburg, for the first time, showed that cancer cells do not follow this principle, since even under normoxic condition they prefer to exploit the glycolytic pathway, producing lactic acid from glucose. Indeed, while normal cells in the presence of oxygen use the oxidative phosphorylation, most cancer cells prefer the glycolysis, a phenomenon termed “Warburg effect” or aerobic glycolysis [2]. This is of a special importance for proliferating cancer cells which may regenerate NAD^+^, increase the availability of glycolytic biosynthetic intermediates and lactate production. Lactate may contribute to sustain proliferation either by stimulating the production of vascular endothelial growth factor or by promoting cellular motility, two favorable aspects for proliferating cancer cells, e.g., generation of new blood vessels and expansion in neighboring tissues [3]. Lactic acid production and its release into the tumor microenvironment helps reduce the local extracellular pH, which might be instrumental for tumor progression, by promoting the invasive abilities of cancer cells [4], their resistance to apoptotic stimuli as well as chemo- and target therapies [5], by inducing anoikis resistance thus favouring tumor cell survival into the circulatory system [6], and importantly, by inhibiting the immune response supporting tumor cell escape [7]. The Warburg phenotype is regulated by numerous oncogenes, e.g., MYC transcription factor has been found to activate lactate dehydrogenase (LDH)A [8], and promote the switch from pyruvate kinase muscle isozyme 1 (PKM1) to 2 (PKM2), a limiting glycolytic enzyme of the final step of glycolysis, involved in the pyruvate and ATP production from phosphoenolpyruvate [9]. PKM2, in its less active dimeric form, reduces ATP generation leading to the production of lactate and several glycolytic intermediates, used as building blocks for the biosynthesis of cellular macromolecules, such as amino acids, lipids and nucleotides. In addition, mammalian target of rapamycin (mTOR) was demonstrated to be a key activator of the Warburg effect, as it induces under normoxic conditions several glycolytic enzymes, including PKM2 [10]. 

Recently, plant-derived compounds have drawn the attention of the scientific community for their several beneficial properties. In particular, polyphenols have been subjected to numerous studies and they showed anti-oxidant, anti-inflammatory, cardio- and neuro-protective functions as well as anti-cancer activity [11,12,13,14,15]. Moreover, their anti-cancer activity has been proved in a broad range of cancer models, so that some of these natural compounds have been included in clinical trials [16,17], as they showed promising effects in terms of promoting the anti-cancer response and decreasing at the same time the toxicity of conventional therapies [18,19,20,21,22]. 

Oleuropein (Ole) is the main bioactive phenolic compound of *Olea europaea* L. that has attracted great interest in the prevention and therapy of several non–communicable diseases, including cancer [23]. As to its anti-cancer properties, Ole affects and modulates multiple different biochemical processes and pathways involved in carcinogenesis. Indeed, Ole exerts an inhibitory effect on cancer cell proliferation, tumor growth and angiogenesis; it reduces inflammation and induces apoptosis [23,24,25]. In our previous study we found that Ole affects both the proliferation and the viability of A375 BRAF melanoma cells and potentiates their therapy response through pAKT/mTOR pathway [26]. In addition, we observed that an olive leaf extract enriched in Ole (OLEO), used at equimolar Ole concentration, was more effective to potentiate the cytotoxic effect, co-administered with conventional chemotherapeutic agents, compared to Ole alone [26]. Following this line of research, we decided to investigate if OLEO could be able to inhibit the metabolism of BRAF melanoma cells, that are usually glycolysis-addicted. The existence of a strong link between tumor-specific signalling pathways and metabolic adaptations is well known. Therefore, interfering with metabolic processes and metabolic enzymes may be a key strategy for cancer therapy. In this context, significant efforts have been recently done to elucidate how plant-derived natural compounds may act as modulators of tumor cell metabolism and, in this way, exert their anti-cancer activity [27].

Gerhauser, revising the knowledge on tumor metabolism and epigenetic variation of glycolytic genes, discovered that several of these processes are influenced by natural compounds [28]. Then, Gao and Chen underlined how several natural compounds may regulate HIF-1α-dependent anaerobic glycolysis of tumor cells: this actually represents a great contribution underlining the ability of natural products to inhibit one of the most critical transcription factors, i.e., HIF-1α, in cancer progression [29]. In this study, we proved that OLEO is able to reduce the glycolytic rate of both primary and metastatic melanoma cells, reducing the expression levels of critical glucose and lactate transporters (glucose transporter-1 (GLUT1) and monocarboxylate transporter-4 (MCT4), respectively) and enzymes, such as PKM2. Extending the study to other tumor types, we observed that OLEO is able to inhibit the glycolytic metabolism also in colorectal, breast and chronic myeloid leukemia cancer cells.

## 2. Results

**Figure 1:**
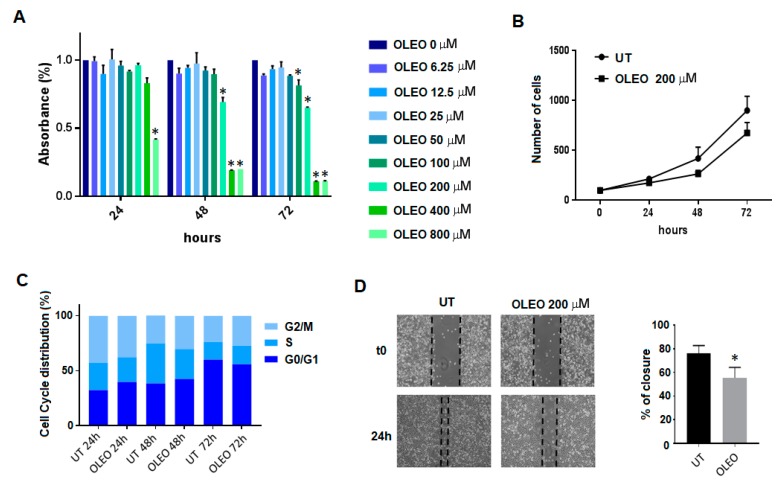
Effects of Ole-enriched leaf extract (OLEO) on A375 melanoma cells. (**A**) Dose-time response evaluated by MTT assay. Significance is indicated with *; (**B**) Cell growth of A375 human melanoma cells treated with OLEO 200 μM; (**C**) Cell cycle distribution analyzed using FACS; (**D**) Effect of OLEO 200 μM on the motility of A375 cells evaluated by scratch wound healing assay. Significance is indicated with *.

In a previous work, with the aim to verify whether Ole might potentiate drug efficiency on BRAF mutant melanoma cells, we decided to use a non-toxic 250 µM dose able to reduce cell proliferation rate without affecting cancer cell viability and apoptosis. We found that Ole potentiates the cytotoxic effect of everolimus against BRAF melanoma cells inhibiting pAKT/mTOR pathway, as measured by the decrease of pAKT/S6. This effect was also demonstrated using an olive leaf extract enriched in an equimolar concentration of Ole [26]. Here, we confirmed that a similar OLEO, at a 200 μM dose, reduces the viability of A375 melanoma cells in a very limited amount (see the 48 and 72 h of treatment), as cell proliferation without modifying cell cycle phase distribution (Figure 1A–C). The same concentration of the extract does not modify viability of human mesenchymal stem cells at each time point of the experiments (see Figure S1). Further, the OLEO, at a 200 μM dose, significantly reduced the closure of a wound (Figure 1D), which was used as an assay of cell motility. The reduced closure of wounds of OLEO-treated melanoma cells discloses the ability of this natural product to inhibit cell motility. These findings prompted to investigate effects of OLEO on melanoma metabolism. We know that V600E mutant BRAF melanoma cells are strictly addicted to glycolysis, the so-called Warburg effect, thus it was possible that a reduction of the glycolytic pathway may have a role in the decreased proliferation and motility of OLEO-treated melanoma cells.

We evaluated the metabolic profile of melanoma cells after 200 μM OLEO administration through Seahorse Bioanalyzer XF96 analysis, thereafter studying the metabolic markers through real time PCR and Western Blot analysis. 

**Figure 2:**
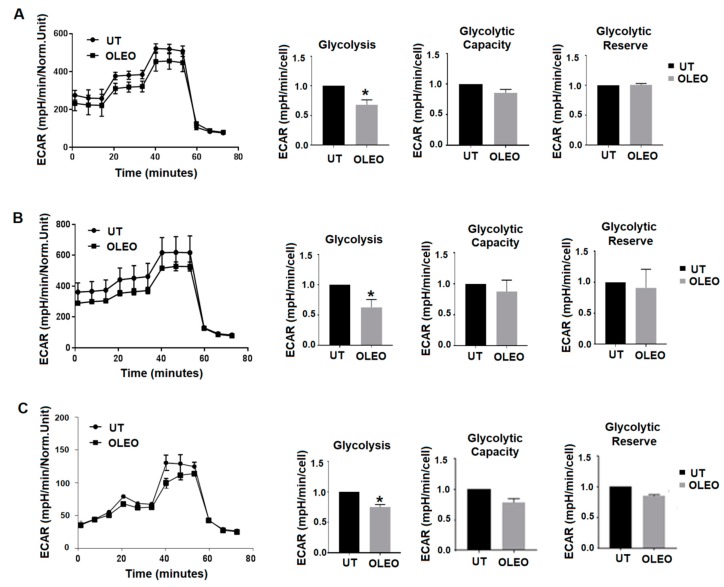
Representative results of a glucose stress test of A375 melanoma cells treated with 200 μM OLEO (**A**) or 200 μM Ole (**B**) for 24 h, and of A375-M6 melanoma cells treated with 200 μM OLEO for 24 h (**C**). Plots on the right represent glycolysis, glycolytic reserve and glycolytic capacity extracted from glycolysis stress assay results obtained using the Seahorse Analyzer. Significance is indicated with *.

We first tested the effect of OLEO extract on the glycolytic activity of A375 melanoma cells using a glycostress standard assay. Overall, OLEO impairs glycolysis rate without modifying glycolytic capacity and reserve of melanoma cells (Figure 2A). On the other hand, Mito stress analysis indicates that OLEO does not modify the respiration of melanoma cells.

To add information on OLEO-driven glycolysis inhibition, we exploited the Seahorse Bioanalyzer XF96 to check the metabolism of A375 melanoma cells exposed for 24 h to an equimolar concentration of pure Oleuropein, and we found an equivalent reducing effect on the glycolysis rate (Figure 2B), without any modification of respiration of these cells. 

Further, to sustain the inhibitory role on the glycolytic metabolism of melanoma cells by OLEO, we tested its glycolysis inhibitory effect on a metastatic clone of A375 melanoma cells, called A375-M6, isolated from the lung of immunodeficient animals. In line with the previous results, we observed that OLEO exerts the same inhibitory effect on glycolysis of these metastatic cells, as showed in the glycostress analysis (Figure 2C). Overall, OLEO is able to repress the aerobic glycolysis of primary and metastatic melanoma cells.

**Figure 3:**
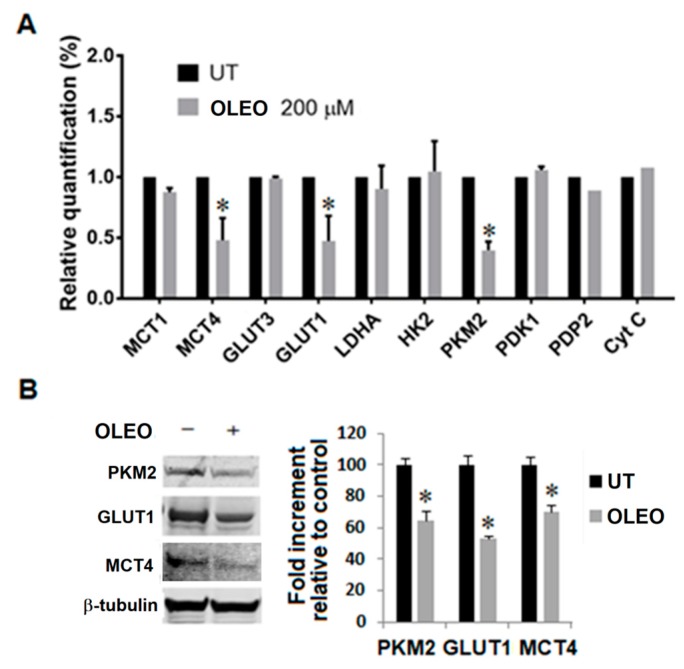
Change in metabolic markers of A375 melanoma cells treated with OLEO 200 μM for 24 h. (**A**) Evaluation by quantitative real-time PCR of genes involved in metabolism; (**B**) Representative Western blot panels of PKM2, GLUT1 and MCT4 protein levels. Each band in the Western blot was quantified by densitometric analysis and the corresponding histogram was constructed by normalizing the density of each band to that of β-tubulin. Values presented are means ± SEM of three independent experiments. Significance is indicated with *.

Along with the dynamic investigation of metabolism expressed by A375 and A375-M6 melanoma cells following OLEO treatment, we identified a series of glycolytic biomarkers down regulated by our nutraceutical product. Testing both mRNA and protein levels, we observed that glucose transporter isoform 1 (GLUT1), pyruvate kinase isozymes M2 (PKM2) and monocarboxylate transporter 4 (MCT4) of OLEO-treated A375 melanoma cells are reduced by a 50% compared to control (i.e., untreated melanoma cells) (Figure 3). To underline the importance of these three key glycolytic biomarkers inhibited by OLEO: (1) GLUT1 is the major glucose transporter in cancer cells; (2) PKM2 is a modulator of glucose metabolism sustaining building block generation needed for cell proliferation; (3) MCT4 exports lactate and protons produced by glycolysis, preventing the inhibition of glycolytic enzymes such as phosphofructokinase activity, that is reduced by intracellular acidification.

**Figure 4:**
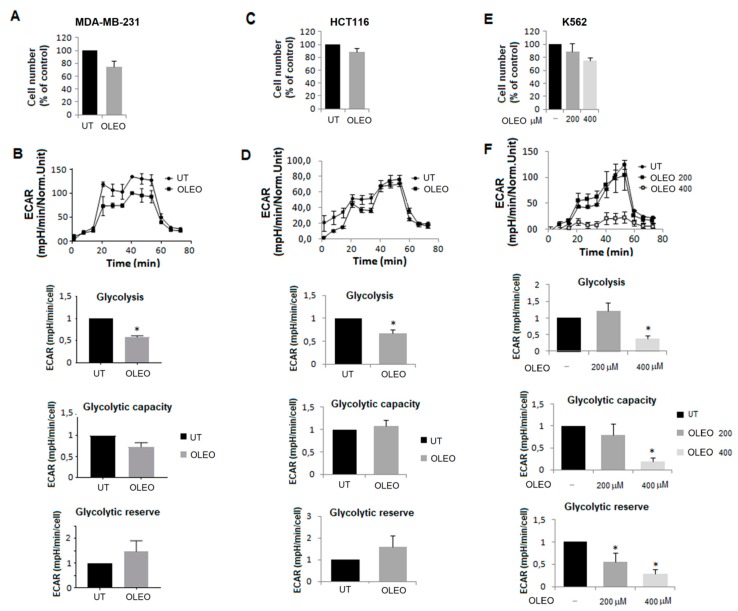
Effect of OLEO on glycolytic metabolism of breast cancer, colon carcinoma and myeloid leukemia cells. Cell number (**A**,**C**,**E**) and representative results of a glucose stress test of MDA-MB-231 (**B**), HCT116 (**D**), and K562 (**F**) cells, treated with 200 or 400 μM OLEO for 48 h. Plots on the right represent glycolysis, glycolytic reserve and glycolytic capacity extracted from glycolysis stress assay results obtained using the Seahorse Analyzer. Significance is indicated with *.

To extend our investigation on OLEO metabolic inhibition in cancer cells, we also tested HCT116 (a human colorectal carcinoma cell line), MDA-MB-231 (an undifferentiated triple-negative breast cancer cell line) and K562 cells (a chronic myeloid leukemia cell line) through the Seahorse Bioanalyzer XF96. OLEO does not modify substantially number and viability of colorectal, breast and leukemia cancer cells (Figure 4A,C,E), but was effective in reducing glycolysis rate of all these type of cancer cells; a higher dose of OLEO was needed to inhibit glycolysis of K562 cells. Overall, these results demonstrate a metabolic inhibitory activity of OLEO on a wide array of cancer histotypes, including that of malignant cells of a clonal disorder of hematopoietic stem cells (Figure 4B,D,F). 

## 3. Discussion

It is well known that cancer cells, compared to normal tissues, are characterized by a high rate of glycolytic metabolism. They indeed prefer to use glycolysis even in the presence of enough oxygen to sustain the oxidative phosphorylation (the so-called “Warburg effect” or aerobic glycolysis, to be distinguished from the anaerobic glycolysis exploited under hypoxic conditions). The higher glycolytic rate of cancer cells ensures them an adequate amount of energy and an ample availability of intermediate macromolecules useful to sustain a rapid cell proliferation and tumor mass expansion [3]. Nowadays, the deregulated metabolism is considered a hallmark of cancer and the identification of new compounds able to modulate tumor metabolism is under intense investigation. For this reason, natural agents can be a great importance, in particular because they demonstrated to interfere with most of the activities of cancer cells, at the same time showing, very low toxic effects on normal cells [11,18,30]. To sum up, several authors have underlined how plant-derived natural products interfere with tumor metabolism [22]. 

Here we show that OLEO is able to exert a significant inhibitory effect on cancer cell glycolysis. In particular, by a dynamic evaluation of cancer cell metabolism through the Seahorse Bioanalyzer XF96 platform, we observed that OLEO reduces the glycolytic rate of primary and metastatic melanoma cells, but also of colorectal, breast and chronic myeloid leukemia cancer cells. In line with our results, Sharma and colleagues showed that morin and/or esculetin impaired glycolysis and glutaminolysis preventing colon carcinogenesis [31]. Moreover, Gomez de Cedron and colleagues identified a new series of polyphenols characterized by a galloyl based “head” and a hydrophobic N-acyl “tail”, able to inhibit glycolysis and mitochondrial respiration in colon cancer cells [32]. 

We observed that the glycolytic reduction exerted by our OLEO in melanoma cells is associated with a decreased GLUT1 expression, at both the mRNA and protein levels. GLUT1 is an important target in cancer treatment, being over-expressed by a wide range of tumor cells. Cancer cells may indeed take great advantages of the GLUT1 rapid response and its high affinity for glucose, in order to overcome the several stress conditions encountered in the host microenvironment and continue the progression towards malignancy. The *K*_M_ value of GLUT1 for glucose is near 1 mM, a significantly less amount compared to the normal glucose level found in serum, allowing a relentless glucose transport into the cells. Of interest, GLUT1 represents the predominant glucose transporter isoform of fetus tissues, which exhibit a higher growth rate than adult ones, at comparable levels to those observed in tumor cells, requiring an increased supply of energy-producing substrates [32]. After birth, GLUT1 expression levels decrease and, even though the reasons behind its decline are not yet clear, it could occur a possible switch form a carbohydrate to a fat source of fuel that may induce this change in some organs [33]. For all these reasons, the development of new clinical strategies involving natural GLUT1 inhibitors such as OLEO, in combination with conventional anticancer agents, deserves the attention of the scientific community, sounding as promising combined therapeutic strategy, as recently reported for other natural compounds [34,35].

Along with GLUT1, we showed that OLEO is also able to down regulate PKM2, one of the four pyruvate kinase isoforms which is highly expressed in rapidly proliferating tissues including cancer. This metabolic enzyme is regulated by oncogenic tyrosine kinases which usually lead to an increase glycolytic rate in tumor cells. Despite, tyrosine phosphorylation of glycolytic enzymes usually increases the activities of a majority of glycolytic enzymes, the tyrosine phosphorylation of PKM2 paradoxically results in a decreased PKM2 activity that in turn promotes the Warburg effect [36]. It is possible that the OLEO-driven PKM2 reduction may reduce its glycolytic promotion [37]. PKM2 overexpression was observed in melanoma human samples compared to naevi, showing a gradient of increased expression from radial growth phase to metastatic melanoma. Furthermore, recent studies have shown that PKM2 is also able to act as a protein kinase using phosphoenolpyruvate as a substrate to promote tumorigenesis [36]. Then, Zhang and colleagues found that miR-625-5p regulates PKM2 expression at both mRNA and protein levels in melanoma cells, disclosing a miR/PKM2 role in glucose metabolism of melanoma cells [38]. PKM2 expression has been shown to be also reduced by other natural products such as resveratrol and curcumin. In particular, resveratrol inhibits aerobic glycolysis and PKM2 enzyme in HeLa (human cervical cancer), HepG2 (human liver cancer) and MCF-7 (human breast cancer) cancer cells through the inhibition of mTOR signaling [39]. Resveratrol was also demonstrated to impair hexokinase-2 enzyme in human non-small cell lung cancer cells inhibiting Akt signaling pathway [40], and pyruvate dehydrogenase complex in colon cancer cells [41]. Curcumin, a further well-known phytopolyphenolic compound, has been shown to decrease glucose uptake and lactate production in several cancer cells (lung, breast, cervical, prostate and embryonic kidney cancer cell lines) down-regulating PKM2 expression, interfering with the mTOR-HIF-1α axis [42]. 

MCT4 is the other glycolytic marker that we found to be inhibited by OLEO in melanoma cells. This monocarboxylate transporter acquires a key role in the metabolic activity of glycolytic cells through the proton-coupled transport of monocarboxylates, such as L-lactate, ketone bodies and pyruvate. An immunohistochemical study of the expression of MCT4 in 356 melanoma-bearing patients revealed that this glycolytic marker is significantly increased in metastatic lesions and associated with a poor prognosis [43].

To conclude, in this study we demonstrated that OLEO is able to reduce the high glycolytic activity of various solid tumors, like melanoma, colorectal and breast cancer, but also of chronic myeloid leukemia cells, suggesting a possible usage of this natural product in combination with conventional therapy for a wide range of malignancy.

## 4. Materials and Methods 

### 4.1. Olive Leaf Extract’s Preparation and Toxicity 

The OLEO used to treat normal and cancer cells was prepared and characterized as previously described ([26], see Figures S3 and S4). *Olea europaea* L. (cultivar Leccino), organic green leaves, were collected in April 2018 in Tuscany (Vinci, Florence, Italy) and immediately processed. The extraction using 15% of *Olea* leaves (45 g leaves/300 g double-distilled and purified water), was performed in water at a temperature of 50 °C for 60 min and at room temperature over the night (12 h) [44]. The final powder is obtained by lyophilization with a LYOVAC GT 2 system (Leybold GmbH, Cologne, Germany), freeze-drying yield 1.85%. The identity of the phenolic compounds of *Olea* dry extract powder and the composition of the solution used for the test in vitro, enriched in oleuropein, was ascertained using data from the HPLC/DAD and HPLC/mass spectrometry analyses, in accordance with a previous paper [45]. 

All the solvents (HPLC grade) and formic acid (ACS reagent) were purchased from Aldrich Chemical Company Inc. (Milwaukee, WI, USA). Tyrosol, luteolin 7-O-glucoside, chlorogenic and Ole were obtained from Extrasynthese S.A. (Genay, France). The HPLC-grade water was obtained via double-distillation and purification with a Labconco Water Pro PS polishing station (Labconco Corporation, Kansas City, MO, USA).

The OLEO used in our study has been tested in a sub-acute test of toxicity (7 days) on female F344 rats fed a diet containing 2.7 g of extract/kg of diet (corresponding to a dosage of 100 mg of extract/kg of b.w.) without inducing any change of body weight. 

This lack of toxicity is in agreement with a recent study of Guex et al., which showed that an olive leaf extract, in vivo tested up to 2000 mg/kg, had no toxic or unwanted effects on rats [46]. Moreover, Sepporta et al. [47]’s paper demonstrated absence of toxicity in mice administered 125 mg of Ole/kg (b.w.). The standard dose of Ole used in vivo animal model, from 10 to 125 mg/kg, did not induce toxic effects, evaluated in terms of viability of the animals [48] or liver biomarkers, such as alanine and aspartate aminotransferase activities [49].

### 4.2. Cell Lines and Culture Conditions

In this study we used A375 human melanoma cell lines, obtained from American Type Culture Collection (ATCC, Rockville, MD, USA), A375M6, isolated in our laboratory from lung metastasis of SCID bg/bg mice i.v. injected with A375 [5]; human colorectal carcinoma cell line HT116, a kind gift of Dr. Matteo Lulli (Department of Clinical and Experimental Biomedical Sciences, University of Florence, Italy); human breast carcinoma cells MDA-MB-231, obtained from American Type Culture Collection (ATCC); human leukemia cells K562, a kind gift of Prof. Persio Dello Sbarba (Department of Clinical and Experimental Biomedical Sciences, University of Florence) and human mesenchymal stem cells (MSC) obtained from bone marrow aspirates of donors which signed informed consent [50]. A375, A375M6, HCT116 and MDA-MB-231 were cultured in Dulbecco’s Modified Eagle Medium high glucose (DMEM 4500, EuroClone, Milan, Italy) supplemented with 10% fetal bovine serum (FBS, EuroClone); K562 were cultured in Roswell Park Memorial Institute 1640 medium (RPMI, EuroClone) supplemented with 10% FBS; MSC were expanded in Dulbecco’s modified Eagle’s medium with low glucose (DMEM 1000; Gibco, Life Technologies, Monza, Italy) supplemented with 20% FBS. Cells were maintained at 37 °C in humidified atmosphere containing 90% air and 10% CO_2_ and they were harvested from subconfluent cultures by incubation with a trypsin-EDTA solution (EuroClone), and propagated every three days. Viability of the cells was determined by trypan blue exclusion test. Cultures were periodically monitored for mycoplasma contamination using Chen’s fluorochrome test. Cells were treated with OLEO for 24–72 h.

### 4.3. MTT Assay

Cell viability was assessed using MTT (3-(4,5-dimethylthiazol-2-yl)-2,5-diphenyltetrazolium bromide) tetrazolium reduction assay (Sigma Aldrich, Milan, Italy) as described in [26]. Cells were plated into 96-multiwell plates in complete medium without red phenol. The treatment was added to the medium colture at different dose and times, according to the experiment. Then the MTT reagent was added to the medium and plates were incubated at 37 °C. After 2 h, MTT was removed and the blue MTT–formazan product was solubilized with dimethyl sulfoxide (DMSO, Sigma Aldrich). The absorbance of the formazan solution was read at 595 nm using the microplate reader (Bio-Rad, Milan, Italy).

### 4.4. Cell Cycle Analysis

Cell cycle distribution was analyzed by the DNA content using propidium iodide (PI) staining method. Cells were centrifugated and stained with a mixture of 50 µg/mL PI (Sigma-Aldrich, St. Louis, MO, USA), 0.1% trisodium citrate and 0.1% NP40 (or triton x-100) in the dark at 4°C for 30 min. The stained cells were analyzed by flow cytometry (BD-FACS Canto, BD-Biosciences, San Jose, CA, USA) using red propidium-DNA fluorescence as previously described [5].

### 4.5. Wound Healing Assay

Cell migration was evaluated by an in vitro wound healing assay as previously described [6]. Cells were treated for 24 h with the extract, then cells have been detached and sown in 35 mm dishes at high confluence; cell monolayer was wounded with a sterile 200 mL pipette tip, washed with PBS and incubated in 1% FBS culture medium. Wound was analyzed following a 24-h incubation and photographed using phase contrast microscopy.

### 4.6. Seahorse Analysis

Seahorse analysis has been performed as previously described [51]. The extracellular acidification rate (ECAR) and the Oxygen Consumption Rate (OCR) were determined using the Seahorse XF96 Extracellular Flux Analyzer (Seahorse Bioscience, Billerica, MA, USA) through Seahorse XF Glycolysis Stress Test Kit (Agilent Technologies, Santa Clara, CA, USA), measuring preferentially the glycolytic function in cells, or Seahorse XF Mito Stress Test Kit (Agilent Technologies), measuring the dependence of cells on the oxidative metabolism. Cells were counted and seeded in XF96 Seahorse^®^ microplates precoated with poly-D-lysine (ThermoFisher Scientific, Waltham, MA, USA). Cells were suspended in XF Assay Medium supplemented with 1 mM glutamine (from EuroClone, Paington, UK) in order to assess ECAR, in XF Assay Medium supplemented with 2 mM glutamine in order to assess OCR. Cells were left to adhere for a minimum of 30 min at 37 °C. The plate was left to equilibrate in a CO_2_-free incubator before being transferred to the Seahorse XF96 analyzer. The pre-hydrated cartridge was filled with the indicated compounds and calibrated for 30 min in the Seahorse Analyzer. All the experiments were performed at 37 °C. Normalization to protein content was performed after each experiment. The Seahorse XF Report Generator automatically calculated the parameters from Wave data that have been exported to Excel or Graphpad. 

### 4.7. RNA Isolation and Quantitative PCR (qPCR)

Total RNA was isolated from cells by using TRI Reagent (Sigma, Milan, Italy). The amount and purity of RNA were determined spectrophotometrically. cDNAwas obtained by incubating 2 μg of total RNA with 4 U/μL of M-MLV reverse transcriptase (Promega, San Luis Obispo, CA, USA) according to the manufacturer’s instructions. Quantitative real time PCR (qPCR) was performed as reported in [51] using the GoTaq^®^ Probe Systems (Promega). The qPCR analysis was carried out in triplicate using an Applied Biosystems 7500 Sequence Detector with the default PCR setting: 40 cycles at 95 °C for 15 s, 60 °C for 60 s. mRNA was quantified with the DDCt method as described [52]. mRNA levels were normalized to β2 microglobulin as an endogenous control. The primer sequences used are listed in Table 1.

### 4.8. Western Blotting Analysis

Cells were lysed and separated using electrophoresis as previously described [5]. Cells were washed with ice cold PBS containing 1 mM Na4VO3, and lysed in 100 mL of cell RIPA lysis buffer (Merck Millipore, Vimodrone, Milan, Italy) containing PMSF (Sigma-Aldrich), sodium orthovanadate (Sigma-Aldrich) and protease inhibitor cocktail (Calbiochem). Aliquots of supernatants containing equal amounts of protein (40 mg) in Laemmli buffer were separated on Bolt^®^ Bis-Tris Plus gels 4e12% precast polyacrylamide gels (Life Technologies, Monza, Italy). Fractionated proteins were transferred from the gel to a PVDF (polyvinylidene difluoride) membrane using iBlot 2 system (Life Technologies, Monza, Italy). Blots were stained with Ponceau red to ensure equal loading and complete transfer of proteins, and then they were blocked for 1 h, at room temperature, with Odyssey blocking buffer (Dasit Science, Cornaredo, Milan, Italy). Subsequently, the membrane was probed at 4 °C overnight with primary antibodies diluted in a solution of 1:1 Odyssey blocking buffer/T-PBS buffer. The primary antibodies were: rabbit anti-PKM2 (1:1000, Cell Signaling Technology, Danvers, MA, USA), rabbit anti-MCT-4 (1:1000, Santa Cruz Biotechnology, Santa Cruz, CA, USA), rabbit anti-GLUT1 (1:1000, Cell Signaling Technology). The membrane was washed in T-PBS buffer, incubated for 1 h at room temperature with goat anti-rabbit IgG Alexa Flour 750 antibody or with goat antimouse IgG Alexa Fluor 680 antibody (Invitrogen, Monza, Italy), and then visualized by an Odyssey Infrared Imaging System (LI-COR^®^ Bioscience, Lincoln, NE, USA). Mouse anti-β tubulin monoclonal antibody (1:1000, Cell Signaling Technology) was used to assess equal amount of protein loaded in each lane.

### 4.9. Statistical Analysis

Densitometric data are expressed as means ± standard errors of the mean (SEM) depicted by vertical bars from representative experiment of at least three independent experiments. Statistical analysis of the data was performed by ANOVA and Tukey’s multiple comparisons test, and *p* ≤ 0.05 was considered statistically significant. 

## 5. Conclusions

The rapid growth of cancer cells mainly depends on their high glycolytic metabolism. Indeed, tumor cells, compared to normal tissues, prefer to exploit the glycolytic pathway even in the presence of sufficient oxygen to sustain the oxidative phosphorylation. This is likely due to the fact that glycolysis guarantees a rapid availability of metabolic intermediates, assuring not only sufficient energy for their survival, but also an efficient production of nucleotides, amino acids and lipids needed to duplicate cell content before mitosis. In this study we show that the natural product OLEO is able to reduce the glycolytic rate of a wide range of solid and liquid tumor cells, without affecting their basal respiration but rather down-regulating the expression of three key effectors of the glycolytic pathway, i.e., GLUT-1, PKM2 and MCT4, likely resulting in a decreased glucose entrance and biomass production. Thus, the inhibition of glycolysis by OLEO acquires a great significance for the targeting of cancer cell growth and expansion. Our findings, together with previous evidence showing the anti-cancer effects exerted by OLEO, pure Ole and analogs [23,53,54], reinforce the hypothesis to propose the use of these natural compounds in combination with conventional therapy used in the treatment of cancer.

## Figures and Tables

**Table 1:** Primer sequences for PCR.

Gene	FW	RV
MCT1	5’-GTGGCTCAGCTCCGTATTGT-3’	5’-GAGCCGACCTAAAAGTGGTG-3’
MCT4	5’-CAGTTCGAGGTGCTCATGG-3’	5’-ATGTAGAGGTGGGTCGCATC-3’
GLUT1	5’-CGGGCCAAGAGTGTGCTAAA-3’	5’-TGACGATACCGGAGCCAATG-3’
GLUT3	5’-CGAACTTCCTAGTCGGATTG-3’	5’-AGGAGGCACGACTTAGACAT-3’
LDHA	5’-AGCCCGATTCCGTTACCT-3’	5′-CACCAGCAACATTCATTCCA-3′
PKM2	5’-CAGAGGCTGCCATCTACCAC-3’	5’-CCAGACTTGGTGAGGACGAT-3’
PDK1	5’-CCAAGACCTCGTGTTGAGACC-3’	5’-AATACAGCTTCAGGTCTCCTTGG-3’
HK2	5’- CAAAGTGACAGTGGGTGTGG-3’	5’- GCCAGGTCCTTCACTGTCTC-3’
18s	5’-CGCCGCTAGAGGTGAAATTCT-3’	5’-CGAACCTCCGA CTTTCGTTCT-3’
PDP2	5’-ACCACCTCCGTGTCTATTGG-3’	5’-CCAGCGAGATGTCAGAATCC-3’
CytC	5’-TTGCACTTACACCGGTACTTAAGC-3’	5’-ACGTCCCCACTCTCTAAGTCCAA-3
GLS1	5’-TGCTACCTGTCTCCATGGCTT-3’	5’-CTTAGATGGCACCTCCTTTGG-3’

## Supplementary Materials

The following are available online at https://www.mdpi.com/2072-6694/12/2/317/s1, Figure S1: Cell growth of human MSC treated with OLEO 200 μM, Figure S2: Detailed information of protein expression analysis by Western blot: (A) Original blot for the Figure 3B, (B) Densitometry and intensity ratio of each band.
