# Supplementary figures and images for: Cancer Glycolytic Dependence as a New Target of Olive Leaf Extract
Source: Cancers (Basel). 2020 Jan 29;12(2):317. doi: 10.3390/cancers12020317 (PMC7072393; doi:10.3390/cancers12020317)

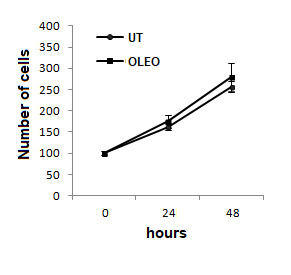

Supplement: Supplementary file 1 [file cancers-12-00317-s001.zip › cancers-686663-supplementary-final/Supplementary Figure S1-Cell growth of human MSC treated with OLEO 200 μM.tif]

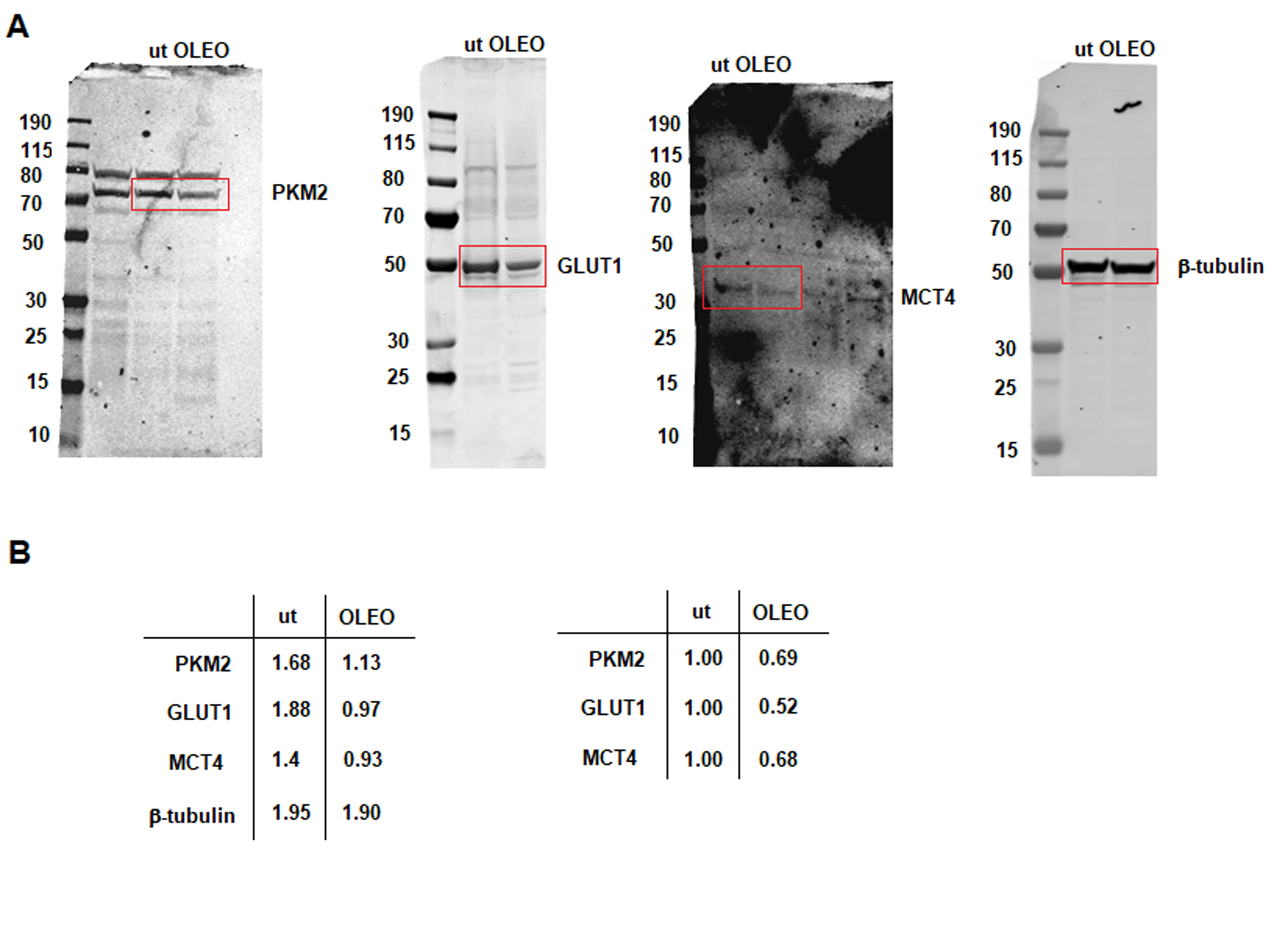

Supplement: Supplementary file 1 [file cancers-12-00317-s001.zip › cancers-686663-supplementary-final/Supplementary Figure S2-Detailed information of protein expression analysis by Western blot (A) Original blot for the Figure 3B, (B) Densitometry and intensity.png]
